# Telomere dysfunction promotes transdifferentiation of human fibroblasts into myofibroblasts

**DOI:** 10.1111/acel.12838

**Published:** 2018-09-22

**Authors:** Neetu Razdan, Themistoklis Vasilopoulos, Utz Herbig

**Affiliations:** ^1^ New Jersey Medical School, Cancer Institute of New Jersey-Newark Rutgers Biomedical and Health Sciences Newark New Jersey; ^2^ Department of Microbiology, Biochemistry and Molecular Genetics Rutgers Biomedical and Health Sciences Newark New Jersey

**Keywords:** hTERT, myofibroblast, SASP, senescence, telomerase, telomere, TGF‐β, transdifferentiation

## Abstract

Cells that had undergone telomere dysfunction‐induced senescence secrete numerous cytokines and other molecules, collectively called the senescence‐associated secretory phenotype (SASP). Although certain SASP factors have been demonstrated to promote cellular senescence in neighboring cells in a paracrine manner, the mechanisms leading to bystander senescence and the functional significance of these effects are currently unclear. Here, we demonstrate that TGF‐β1, a component of the SASP, causes telomere dysfunction in normal somatic human fibroblasts in a Smad3/NOX4/ROS‐dependent manner. Surprisingly, instead of activating cellular senescence, TGF‐β1‐induced telomere dysfunction caused fibroblasts to transdifferentiate into α‐SMA‐expressing myofibroblasts, a mesenchymal and contractile cell type that is critical for wound healing and tissue repair. Despite the presence of dysfunctional telomeres, transdifferentiated cells acquired the ability to contract collagen lattices and displayed a gene expression signature characteristic of functional myofibroblasts. Significantly, the formation of dysfunctional telomeres and downstream p53 signaling was necessary for myofibroblast transdifferentiation, as suppressing telomere dysfunction by expression of hTERT, inhibiting the signaling pathways that lead to stochastic telomere dysfunction, and suppressing p53 function prevented the generation of myofibroblasts in response to TGF‐β1 signaling. Furthermore, inducing telomere dysfunction using shRNA against TRF2 also caused cells to develop features that are characteristic of myofibroblasts, even in the absence of exogenous TGF‐β1. Overall, our data demonstrate that telomere dysfunction is not only compatible with cell functionality, but they also demonstrate that the generation of dysfunctional telomeres is an essential step for transdifferentiation of human fibroblasts into myofibroblasts.

## 1 INTRODUCTION

Cellular senescence is a state of persistent proliferative inactivity evoked by cell extrinsic and intrinsic stresses including telomere dysfunction. Aside from functioning to suppress malignant cancer development, cellular senescence has been demonstrated to play important roles also in other biological processes such as aging, embryonic development, and wound healing (Munoz‐Espin & Serrano, 2014). During tissue repair and wound healing, senescent cells transiently accumulate at the site of injury, where they are thought to limit fibrosis (Jun & Lau, 2010; Krizhanovsky et al., 2008), promote transdifferentiation of fibroblasts into myofibroblasts (Demaria et al., 2014), and attract immune modulatory cells that eventually eliminate newly generated senescent cells at the site of injury (Krizhanovsky et al., 2008). Many of these functions are mediated in a paracrine manner and by a complex mixture of factors that are secreted from senescent cells, collectively called the senescence‐associated secretory phenotype or SASP.

Although numerous stresses can cause cells to enter a senescent state, a SASP containing factors involved in tissue remodeling and repair has been detected only in cells that had undergone senescence due to activation of a persistent DNA damage response (DDR) (Malaquin, Martinez, & Rodier, 2016; Rodier et al., 2009). In these cells, the SASP develops dynamically and changes its composition in a temporal manner. Within a few hours after entering senescence, the secretome of human somatic fibroblasts is enriched in growth factors and includes transforming growth factor beta‐1 (TGF‐β1) (Hoare et al., 2016; Hubackova, Krejcikova, Bartek, & Hodny, 2012), a cytokine involved in wound healing where it promotes transdifferentiation of fibroblasts into myofibroblasts. After approximately four to six days in senescence, the TGF‐β1‐rich secretome changes as cells begin to secrete pro‐inflammatory cytokines that include interleukin 6 (IL‐6) and 8 (IL‐8) (Hoare et al., 2016). While it is still unclear why the SASP develops in phases, it has been suggested that the temporal evolution of the SASP may be important for orchestrating the distinct stages of tissue repair (Hoare & Narita, 2016; Schmitt, 2016).

Surprisingly, conditioned medium from senescent fibroblasts, as well as a number of molecules contained within this secretome including TGF‐β1 and IL1, can cause cellular senescence in neighboring fibroblasts through paracrine mechanisms (Acosta et al., 2013; Hubackova et al., 2012; Nelson et al., 2012). This bystander senescence is a consequence of cytokine‐mediated production of reactive oxygen species (ROS), which ultimately cause the formation of DSBs and activation of a persistent DDR in neighboring cells (Hubackova et al., 2012; Nelson et al., 2012). Thus, the activation of a persistent DDR not only initiates secretion of SASP molecules from a damaged cell, it also activates a persistent DDR in cells that reside in close proximity to this cell in a paracrine fashion. The manner in which cytokine signaling‐generated ROS activate this DDR and cellular senescence in normal human fibroblasts, however, is not known.

One cause of a persistent DDR is telomere dysfunction. In mammalian cells, telomeres consist of kilobases of repetitive TTAGGG sequences that, together with a six‐subunit protein complex called shelterin, form a protective structure at chromosome ends. In cells that lack high levels of hTERT expression, such as somatic human fibroblasts, telomeres are incompletely replicated and therefore shorten with every cell division cycle. Progressive telomere erosion eventually generates one or more telomeres that are so short that the protective structure can no longer be formed. This causes telomeres to become dysfunctional which activates a persistent DDR and promotes cells to enter replicative senescence (d’Adda di Fagagna et al., 2003; Herbig, Jobling, Chen, Chen, & Sedivy, 2004). Significantly, telomere dysfunction‐induced senescence (TDIS) can also be triggered in the absence of continuous cell proliferation and in the absence of critical telomere shortening. For example, genotoxic stresses that cause DSB formation in telomeric repeats can rapidly activate TDIS, as telomeric breaks resist DNA repair activities (Fumagalli et al., 2012; Hewitt et al., 2012; Suram et al., 2012). Stresses that have been demonstrated to cause TDIS include oncogene expression (Patel, Suram, Mirani, Bischof, & Herbig, 2016; Suram et al., 2012), DNA replication stress (Boccardi et al., 2015; Fumagalli et al., 2012; Hewitt et al., 2012), and reactive oxygen species (ROS) (Boccardi et al., 2015), among others. Whether generated due to progressive telomere erosion or due to DSB formation in telomeric repeats, telomere dysfunction activates a persistent DDR that is mediated by and dependent on p53, a transcription factor that not only trans‐activates DDR genes (Molchadsky, Rivlin, Brosh, Rotter, & Sarig, 2010), but also regulates a wide range of other biological activities including cell differentiation (Rivlin, Koifman, & Rotter, 2015).

The myofibroblast is a mesenchymal cell type that performs important functions during wound healing (Tomasek, Gabbiani, Hinz, Chaponnier, & Brown, 2002). In addition to producing components of the extracellular matrix (ECM), including collagens, fibronectin, proteoglycans, and others, myofibroblasts contract and provide rigidity to the connective tissue surrounding the wound. Myofibroblasts are characterized and identified by the de novo expression and incorporation of smooth muscle α‐actin (αSMA) into stress fibers, a feature that is critical for myofibroblast contracture. While myofibroblasts have multiple origins, they are primarily generated from resident fibroblasts at the site of injury by a process called transdifferentiation (Hinz, 2007). The primary factor that initiates fibroblast to myofibroblast transdifferentiation in humans is TGF‐β1, the cytokine that, paradoxically, has also been demonstrated to induce bystander senescence in fibroblasts. Although these observations seem contradictory, they also raise the possibility that transdifferentiation and cellular senescence share a common pathway.

In this study, we demonstrate that TGF‐β1, a component of the SASP, causes telomere dysfunction, proliferative arrest, and myofibroblast transdifferentiation when added to the culture medium of somatic human fibroblasts. Surprisingly, both the formation of dysfunctional telomeres and the resulting activation of p53 were necessary for rapid generation of myofibroblasts, as suppressing telomere dysfunction, inhibiting the pathways that lead to telomere dysfunction, or suppressing p53 signaling prevented transdifferentiation. Furthermore, inducing telomere dysfunction in the absence of exogenous TGF‐β1 also promoted the development of αSMA‐expressing cells, overall demonstrating that telomere dysfunction is a critical event during transdifferentiation of fibroblasts into myofibroblasts in humans.

## 2 RESULTS

### 2.1 The SASP promotes telomere dysfunction in a paracrine manner

**Figure 1:**
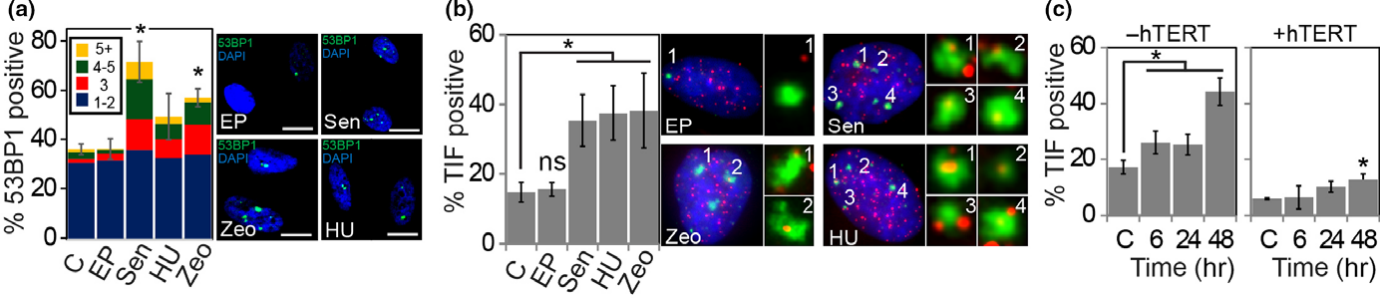
Senescence‐associated secretory phenotype factors cause telomere dysfunction in a paracrine manner in cells that lack hTERT expression. (a) Percentage of early passage human BJ fibroblasts that immunostained positive for 1–2 (blue bars), 3 (red bars), 4–5 (green bars), or more than 5 (yellow) 53BP1 foci after a 48 hr incubation with conditioned medium from early passage cells (EP), senescent cells (Sen), hydroxyurea treated cells (HU), and zeocin treated cells (Zeo). Control cells (C) were untreated. Representative micrographs illustrating 53BP1 (green) foci are shown to the right. Nuclear DNA was counterstained with DAPI (blue). Scale bars: 20 µm. At least 100 cells were analyzed for each experiment. Error bars: ±*SD*. *p < 0.05 (*n* = 4). (b) Percentage of cells that immunostained positive for telomere dysfunction‐induced DNA damage foci (TIF) treated as in (a). Representative micrographs of TIF‐positive cells and telomere (red)‐53BP1 (green) colocalizations for each group are shown to the right. Error bars: ±*SD*. *p < 0.05. (c) Percentage of normal human BJ fibroblasts (−hTERT; left graph) and BJ fibroblasts overexpressing hTERT (+hTERT; right graph) positive for TIF as treated in B. Error bars: ±*SD*. *p < 0.05 (*n* = 3)

Molecules secreted from damaged and senescent cells can activate a persistent DDR in neighboring cells through paracrine mechanisms. As dysfunctional telomeres are a source of a persistent DDR, we tested whether these molecules can cause telomere dysfunction in a paracrine manner. For this, we collected conditioned medium from human BJ fibroblasts that were serially passaged in culture until they entered senescence (Sen), from fibroblasts that were stimulated to undergo senescence upon treatment with the drugs hydroxyurea (HU) and zeocin (Zeo), as well as from early passage BJ cells (EP) as control. Consistent with previous studies (Hubackova et al., 2012), conditioned medium from senescent cells and from drug‐treated cells, but not from early passage cells, induced a DDR in early passage BJ fibroblasts, as demonstrated by a significant increase in the percentage of cells with more than two 53BP1 DDR foci 48 hr following treatment (Figure 1a). Induction of the DDR was dependent on cell proliferation as contact inhibited cells did not develop DDR foci following incubation with senescent cell conditioned medium (Supporting Information Figure S1a). To determine whether these DDR foci were telomere dysfunction‐induced DNA damage foci (TIF), we simultaneously immunostained cells using antibodies against 53BP1 and visualized telomeres by fluorescence in situ hybridization (FISH) using a peptide nucleic acid (PNA) that is complementary to telomeric TTAGGG repeats. We discovered that the majority of 53BP1 foci indeed colocalized with telomeric signals, resulting in a greater than twofold increase in the percentage of cells that were positive for TIF (Figure 1b). Similarly, mean 53BP1‐telomere colocalizations increased by a factor of 2 in response to SASP factors (Figure S1b). Treating early passage BJ fibroblasts with conditioned medium from senescent cells for increasing periods of time revealed that DDR foci and TIF were generated rapidly in response to SASP factors, reaching maximum levels just 48 hr following treatment (Figure 1c and Supporting Information Figure S1c,d). At later times, TIF‐positive cells again declined in abundance, likely due to continued proliferation of cells that were insensitive to SASP factors (Supporting Information Figure S1d). Surprisingly, formation of dysfunctional telomeres was diminished in fibroblasts that overexpressed the catalytic subunit of telomerase, hTERT, at all time points tested, suggesting that telomerase protects telomeres from becoming dysfunctional in response to paracrine signals (Figure 1c and Supporting Information Figure S1c,e). Nontelomeric DDR foci were also reduced in hTERT‐expressing cells, albeit at significantly reduced frequency compared to telomeric DDR foci (Supporting Information Figure S1f), suggesting that hTERT either promotes repair also of nontelomeric double‐stranded DNA breaks, or that hTERT reduces genotoxic stresses that can damage nontelomeric sites. Overall, these data reveal that certain factors secreted from senescent and drug‐treated cells can rapidly promote telomere dysfunction in neighboring cells that lack high levels of telomerase through paracrine signaling mechanisms.

### 2.2 TGF‐β1 causes telomere dysfunction and myofibroblast transdifferentiation in normal human fibroblasts, but not in fibroblasts that express hTERT

**Figure 2:**
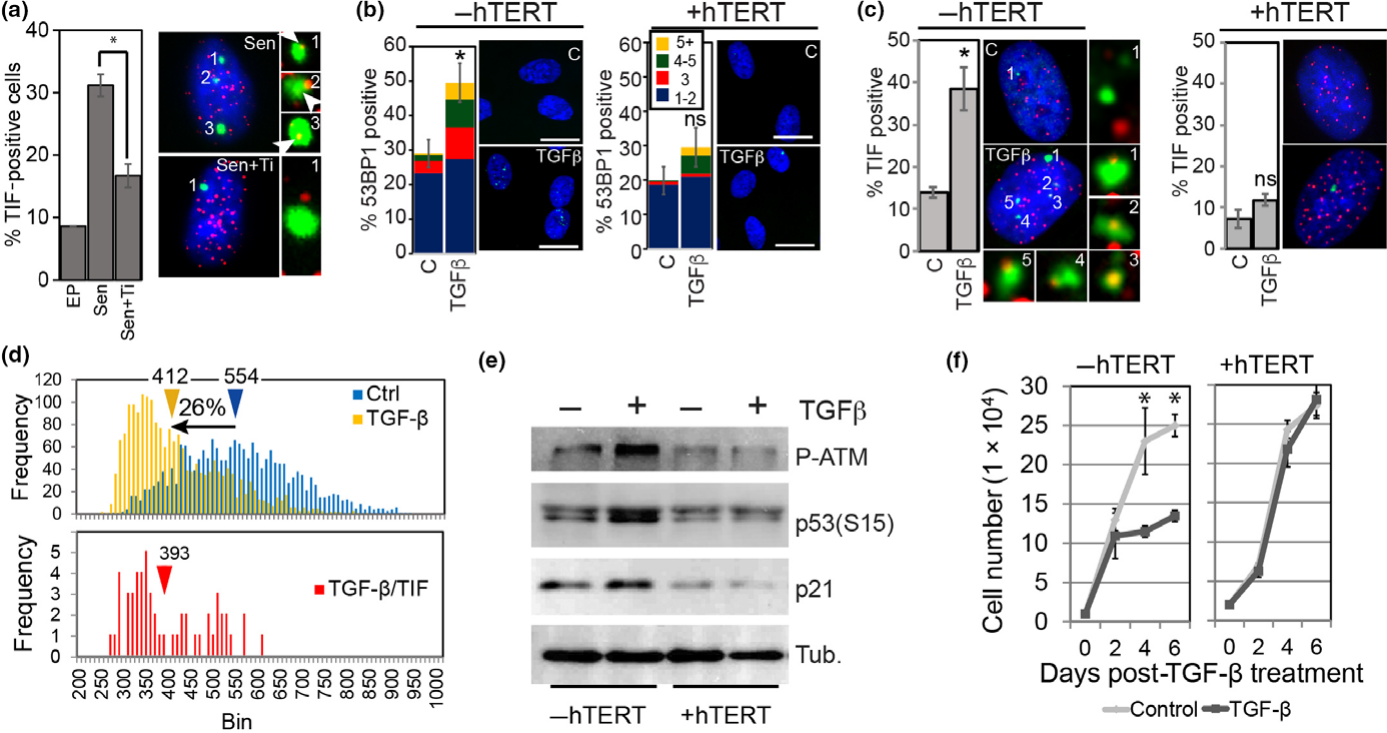
TGF‐β1 causes telomere dysfunction and G1 DNA damage checkpoint activation in normal human fibroblasts. (a) Percentage of early passage BJ fibroblasts that were positive for TIF following treatment with conditioned medium collected from either early passage BJ fibroblasts (EP) or senescent cells (Sen.) in the absence or presence of SB431542, a pharmacological inhibitor of TGF‐β1 signaling for 48 hr (Sen+Ti). Error bars: ±*SD*; (*n* = 4). *p < 0.005. Representative micrographs of TIF‐positive cells and telomere (red)‐53BP1 (green) colocalizations (arrows) for Sen and Sen+Ti group are shown to the right. (b) Percentage of early passage human BJ fibroblasts (−hTERT) and BJ fibroblasts overexpressing hTERT (+hTERT) that immunostained positive for 1–2 (blue bars), 3 (red bars), 4–5 (green bars), or more than 5 (yellow) 53BP1 foci and that were either untreated as control (C) or treated with recombinant TGF‐β1 (10 ng/ml) for 48 hr. Error bars: ±*SD*. *p < 0.05; ns: not significant; (*n* = 3). Representative micrographs illustrating 53BP1 (green) foci are shown to the right. Nuclear DNA was counterstained with DAPI (blue). Scale bars: 20 µm. (c) Percentage of early passage human BJ fibroblasts (−hTERT) and BJ fibroblasts overexpressing hTERT (+hTERT) that immunostained positive for TIF foci and that were treated as in A. Error bars: ±*SD*. *p < 0.005; ns: not significant, *p* > 0.05, (*n* = 3). Representative micrographs of TIF‐positive cells and telomere (red)‐53BP1 (green) colocalizations for (indicated by numbers) are shown to the right. (d) Distribution of telomere lengths based on their signal intensities (*x*‐axis; arbitrary units). Top histogram: all telomeric signals in cells treated with (yellow bars) or without TGF‐β1 (blue bars) for 48 hr. Bottom histogram: telomeric signals associated with 53BP1 foci in cells treated with TGF‐β1 (red bars) for 48 hr. *n* = 1,948, no. of telomeric signals analyzed. Average telomere signal intensities, and percent reduction in response to TGF‐β1 treatment, are indicated. (e) Immunoblots of extracts from early passage BJ fibroblasts (−hTERT) and BJ fibroblasts overexpressing hTERT (+hTERT) that were either control treated (C) or treated with TGF‐β1 for 72 hr using indicated antibodies. β‐tubulin was used as loading control. (f) Proliferation curves of BJ fibroblasts (−hTERT; left graph) and BJ fibroblasts overexpressing hTERT (+hTERT; right graph) following treatment with TGF‐β1 (dark line) or control (light line). Error bars: ±*SD*, *p < 0.005, (*n* = 3)

The SASP is comprised of numerous factors that include pro‐inflammatory cytokines, growth factors, proteases, and also many yet uncharacterized molecules. One component of the SASP that is capable of triggering a DDR in a paracrine manner is TGF‐β1, raising the possibility that signaling through the TGF‐β receptor causes a DDR by promoting telomere dysfunction (Dickey et al., 2009; Hubackova et al., 2012; Kojima, Kunimoto, Inoue, & Nakajima, 2012). Indeed, addition of a specific inhibitor of the TGF‐β receptor I to the culture medium inactivated the telomere dysfunction‐inducing activity of the SASP, suggesting that TGF‐β1 contained within the SASP causes telomere dysfunction in a paracrine manner (Figure 2a). To directly test whether TGF‐β1 activates a DDR by promoting telomere dysfunction, we added recombinant TGF‐β1 to the culture medium and monitored formation of DDR foci and TIF by immunofluorescence and telomere immunoFISH, respectively. Addition of this cytokine indeed triggered a DDR and a greater than twofold increase in TIF‐positive cells and mean 53BP1‐telomere colocalizations within 48 hr of treatment (Figure 2b,c and Supporting Information Figure S2a). Similar to SASP factors that did not cause telomere dysfunction in hTERT overexpressing fibroblasts, TGF‐β1 also did not activate a DDR in BJ fibroblasts that expressed hTERT nor did it promote the formation of TIF in these cells (Figure 2b,c and Supporting Information Figure S2a). Surprisingly, although telomeres in TGF‐β1‐induced TIF were not significantly shorter compared to functional telomeres in the same cells, treating cells with TGF‐β1 for 48 hr caused a 26% reduction of total telomere lengths compared to control treated cells (Figure 2d).

As telomere dysfunction causes activation of a persistent DDR and cellular senescence in normal human fibroblasts (Takai, Smogorzewska, & Lange, 2003), we tested whether TGF‐β1 also leads to DNA damage checkpoint activation and growth arrest in human cells. BJ fibroblasts incubated with recombinant TGF‐β1 displayed high levels of G1 DNA damage checkpoint markers p‐ATM(S1981), P‐p53(S15), and p21, 72 hr after treatment (Figure 2e and Supporting Information Figure S2b). This was in contrast to hTERT‐expressing fibroblasts that lacked high expression of these markers following treatment with TGF‐β1 (Figure 2e and Supporting Information Figure S2b). Similarly, while normal BJ fibroblast cultures displayed dramatically reduced proliferation rates 48 hr following TGF‐β1 treatment, hTERT‐expressing fibroblasts were insensitive to this treatment and continued to proliferate at rates that were similar to control cultures as demonstrated by cell proliferation curves and EdU incorporation assays (Figure 2f and Supporting Information Figure S2c). Our data therefore demonstrate that TGF‐β1 causes rapid telomere erosion, telomere dysfunction, G1 DNA damage checkpoint activation, and a proliferative arrest in normal human fibroblasts, but not in fibroblasts that express hTERT.

**Figure 3:**
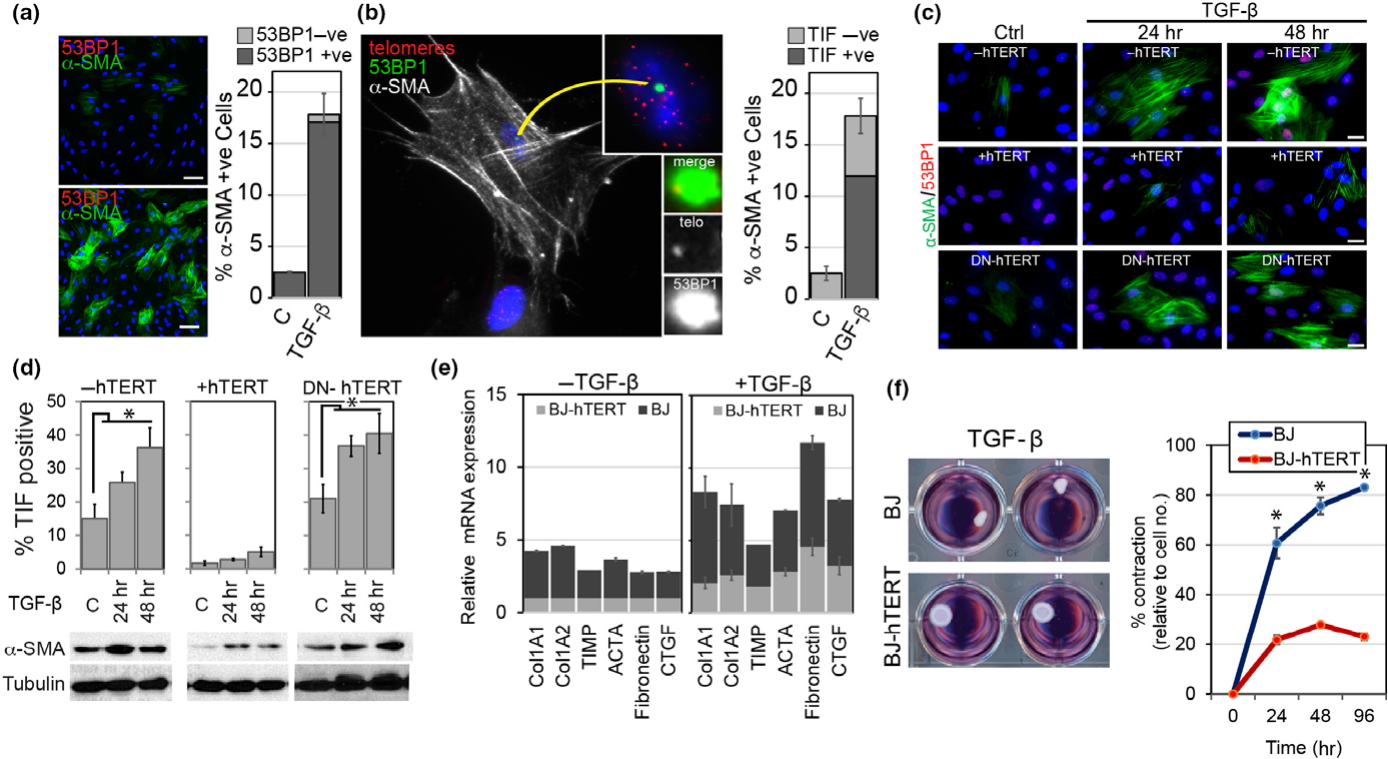
hTERT suppresses TGF‐β1‐induced telomere dysfunction and myofibroblast transdifferentiation. (a) Representative micrographs of control treated (top) and TGF‐β1 treated BJ fibroblasts immunostained for α‐SMA (green) and 53BP1 (red). Cell nuclei were counterstained with DAPI (blue). Bar graph: percentage of α‐SMA‐positive BJ fibroblasts that stain positive (dark gray) or negative (light gray) for at least one 53BP1 focus following treatment with TGF‐β1 (10 ng/ml) or control (C) for 48. Error bars: ±*SE*. *p < 0.05, (*n* = 3). Scale bars: 20 μm. (b) Representative micrograph of TGF‐β1 treated BJ fibroblasts immunostained with using antibodies against α‐SMA (white), 53BP1 (green), and a telomeric FISH probe (red). DAPI: (blue). Insets: enlarged area showing indicating nucleus (top) and 53BP1/telomere colocalizations (bottom). Bar graph: percentage of α‐SMA‐positive BJ fibroblasts that were TIF positive (dark gray) or TIF negative (light gray) following treatment with TGF‐β1 (10 ng/ml) or control (C) for 48. Error bars: ±*SE*. *p < 0.05, (*n* = 3). (c) Immunofluorescence staining of early passage BJ fibroblasts (top row), BJ fibroblasts expressing hTERT (+hTERT; center row) and BJ fibroblasts expressing a dominant defective mutant of hTERT (DN‐hTERT; bottom row) treated with TGF‐β1 (10 ng/ml) for indicated times using antibodies against α‐SMA (green) and 53PB1 (red). Nuclear DNA was counterstained with DAPI (blue). Scale bars: 20 µm. (d) Normal BJ fibroblasts (−hTERT; left panel), BJ fibroblasts overexpressing hTERT (+hTERT; center panel), and BJ fibroblasts overexpressing DN‐hTERT (right panel) were treated with TGF‐β1 (10 ng/ml) and solvent control (C) for indicated time points and analyzed by immunoFISH for percentage of TIF‐positive cells (graphs) and by immunoblotting for α‐SMA expression (immunoblots). γ‐tubulin was used as loading control. Error bars: ±*SD*. *p < 0.05; ns: not significant: *p* > 0.05 (*n* = 3). (e) qRT–PCR analysis of characteristic myofibroblastic genes in the absence of TGB‐β1 treatment (left graph) and following TGB‐β1 treatment for 24 hr (right graph) in normal human BJ fibroblasts (dark bars) and BJ fibroblasts overexpressing hTERT (light bars). Error bars: ±*SD*, *p* < 0.05 comparison between ±hTERT for all genes. (f) Fibroblast populated collagen contraction (FPCL) assay. 4 representative wells containing collagen lattices (white disks) contracted by TGF‐β‐treated BJ cells (top row) and BJ cells overexpressing hTERT (bottom row) are shown in left images. Normal BJ fibroblasts (blue line) and hTERT‐expressing BJ fibroblasts (red line) were mixed with rat tail collagen in the presence of TGF‐β1 for 48 hr. Subsequently, lattices were released and allowed to contract freely for indicated times. Graph illustrates percentage decrease in FPCL area, normalized to cell numbers and measured at indicated times following release. Error bars: ±*SD*. *p < 0.05; (*n* = 3)

Our results demonstrating that TGF‐β1 causes telomere dysfunction through paracrine mechanisms were surprising given that this cytokine, when added directly to the medium of fibroblast cultures, causes these cells to transdifferentiate into myofibroblasts (Desmouliere, Geinoz, Gabbiani, & Gabbiani, 1993). Yet, at the same time our results are also consistent with studies demonstrating that myofibroblasts are nonproliferating cells that are arrested due to G1/S checkpoint activation (Petrov et al., 2008). To test whether TGF‐β1‐treated BJ fibroblasts with dysfunctional telomeres display characteristics of myofibroblasts, we immunostained treated cells using antibodies against 53BP1 together with antibodies against α‐SMA, a molecular marker of fully differentiated myofibroblasts (Hinz, 2007). In contrast to untreated BJ fibroblasts that expressed low levels of α‐SMA, we indeed observed a significant increase in cells that expressed α‐SMA in stress fibers following TGF‐β1 treatment, suggesting that BJ fibroblasts transdifferentiated into myofibroblasts (Figure 3a). Similar results were observed in other human fibroblast strains including GM21, LF1, and WI38 cells (Supporting Information Figure S3a). The great majority of cells with prominent α‐SMA expression also displayed discrete DDR foci (over 95%; Figure 3a), suggesting a causal link between DDR induction and myofibroblast transdifferentiation, as reported previously (Dimitrijevic‐Bussod, Balzaretti‐Maggi, & Gadbois, 1999; Mellone et al., 2016; Sampson et al., 2011). However, since not all cells with DDR foci expressed high levels of α‐SMA in stress fibers (Supporting Information Figure S3d), our data also suggested that activation of the DDR alone is not sufficient for myofibroblast transdifferentiation and that other factors, such as timing of DDR induction or long‐term persistence of the DDR, are critical for activating the transdifferentiation program. As dysfunctional telomeres activate a persistent DDR while nontelomeric DSBs are repaired within minutes of being generated (Fumagalli et al., 2012; Hewitt et al., 2012), we tested whether fibroblasts are stimulated to transdifferentiate preferentially due to a persistent telomeric DDR. By simultaneously immunostaining TGF‐β1‐treated cells for α‐SMA expression and TIF formation, we discovered that the great majority of α‐SMA‐expressing myofibroblasts indeed displayed dysfunctional telomeres and were TIF positive (75%). Our data therefore demonstrate that the myofibroblast‐associated DDR is primarily due to telomere dysfunction.

In order to determine whether the formation of dysfunctional telomeres was necessary for fibroblasts to transdifferentiate into myofibroblasts, we suppressed telomere dysfunction in response to TGF‐β1 signaling by overexpressing hTERT in BJ cells and monitored TIF formation and α‐SMA expression by immunofluorescence analysis and immunoblotting. While normal fibroblasts treated with TGF‐β1 developed dysfunctional telomeres and upregulated α‐SMA expression in a time‐dependent manner, hTERT‐expressing fibroblasts did not develop TIF and showed diminished α‐SMA expression (Figure 3c,d and Supporting Information Figure S3e). Similarly, using human fibroblasts in which dysfunctional telomeres can be repaired using a doxycycline inducible hTERT expression system (Patel et al., 2016), we demonstrate that induction of hTERT expression in TGF‐β1 transdifferentiated myofibroblasts again reduced the levels of α‐SMA expressed in cells (Supporting Information Figure S3f). While the primary function of telomerase is to elongate telomeres and suppress formation of dysfunctional telomeres generated due to telomere shortening and ROS‐induced telomeric replication stress, (Boccardi et al., 2015; Patel et al., 2016), telomerase also possesses noncanonical functions that promote DNA repair and cell survival (Martinez & Blasco, 2011). To determine whether noncanonical functions of hTERT contribute to the suppression of myofibroblast transdifferentiation, we overexpressed in human BJ fibroblasts DN‐hTERT, a dominant defective mutant of hTERT that inactivates the catalytic activity of hTERT while leaving its noncanonical functions intact (Hahn et al., 1999). In contrast to cells that expressed wt hTERT, fibroblasts expressing DN‐hTERT rapidly developed dysfunctional telomeres and upregulated α‐SMA expression in stress fibers in response to TGF‐β1 signaling in a manner similar to normal human fibroblasts (Figure 3c,d and Supporting Information Figure S3e). Our data thus demonstrate that it is the ability of telomerase to elongate short and/or dysfunctional telomeres, and not its noncanonical functions, that suppresses myofibroblast transdifferentiation and that the formation of dysfunctional telomeres is necessary for human fibroblast to develop characteristics of myofibroblasts.

In addition to expressing α‐SMA in stress fibers, myofibroblasts produce collagens, fibronectin, metalloproteinases, and other factors, in order to facilitate remodeling of connective tissue during tissue repair. Equally important for remodeling is that myofibroblasts develop a contractile activity, which is necessary for tissue remodeling (Hinz et al., 2012). To determine whether TGF‐β1‐induced telomere dysfunction in BJ fibroblasts produces functional myofibroblasts capable of contracture, we measured mRNA expression of a number of genes involved in transdifferentiation by qRT–PCR and analyzed contractile activity of TGF‐β1‐treated cells using a fibroblast populated collagen lattice (FPCL) contraction assay. As expected, treating early passage BJ fibroblasts with TGF‐β1 caused cells to upregulate expression of a number of myofibroblastic genes and stimulated their contractile activity in a time‐dependent manner (Figure 3e,f). Significantly, hTERT‐expressing fibroblasts not only displayed reduced expression levels of these genes, but they were also substantially less efficient in contracting collagen lattices (Figure 3e,f). Collectively, our data demonstrate that hTERT suppresses myofibroblast transdifferentiation due to its ability to suppress telomere dysfunction in response to TGF‐β1 signaling.

Our data suggest that telomere dysfunction mediates paracrine signaling to downstream effectors that promote fibroblast to myofibroblast transdifferentiation. This raises the possibility that dysfunctional telomeres in replicatively senescent cells also cause fibroblasts to transdifferentiate into myofibroblasts. Surprisingly, however, expression levels of α‐SMA were substantially reduced in fibroblasts that were passaged into replicative senescence, compared to early passage fibroblasts (Supporting Information Figure S3g,h). In addition, senescent fibroblasts were insensitive to TGF‐β1‐treatment and did not upregulate expression of myofibroblastic genes such as α‐SMA and collagens when stimulated with TGF‐β1 (Supporting Information Figure S3i). This not only demonstrates that replicatively senescent fibroblasts are not myofibroblasts, but also that senescent cells have lost the ability to transdifferentiate into myofibroblasts when stimulated by paracrine signals. Similarly, fibroblasts in oncogene‐induced senescence, a response that is stabilized due to telomere dysfunction (Suram et al., 2012), did not display characteristics of myofibroblasts (Supporting Information Figure S3j). However, senescent cells secrete TGFβ1 capable of inducing α‐SMA expression in a paracrine manner, as a specific inhibitor of TGF‐β receptor I suppressed the ability of senescent cell conditioned medium to induce α‐SMA expression in human fibroblasts (Supporting Information Figure S3k). These data suggest that senescent cells can promote myofibroblast transdifferentiation through their SASP, which is consistent with a recent study (Schafer et al., 2017).

### 2.3 TGFβ1 causes telomere dysfunction and myofibroblast transdifferentiation through SMAD3/NOX4‐dependent ROS production

**Figure 4:**
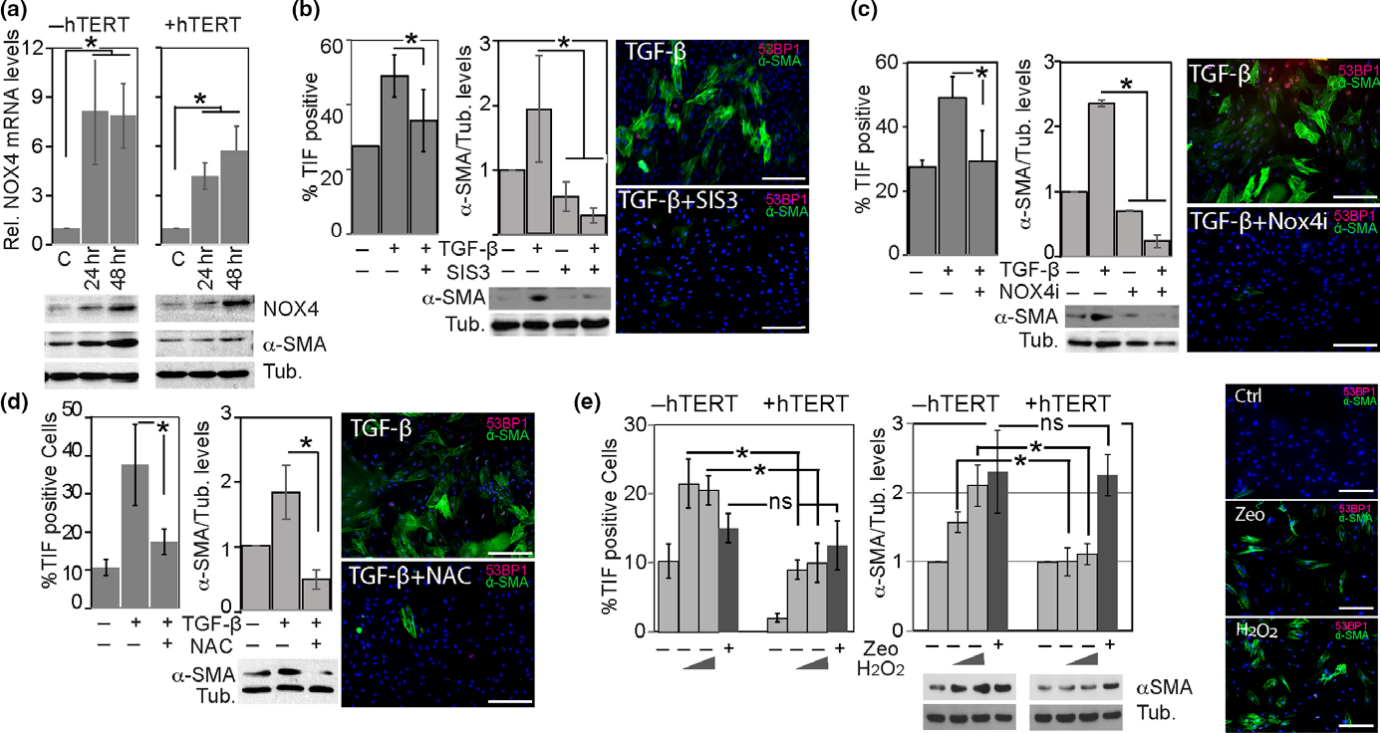
TGF‐β1‐induced transdifferentiation is mediated by Smad3/NOX4‐dependent ROS production and telomere dysfunction. (a) Normal BJ fibroblasts (−hTERT; left graph) and BJ fibroblasts overexpressing hTERT (+hTERT; right graph) were either control treated (C) or treated with TGF‐β12 for indicated times. NOX4 mRNA levels relative to controls, which were set to 1, were measured by qRT–PCR. Error bars: ±*SD*. *p < 0.05, (*n* = 3). Immunoblots below graphs illustrate NOX4 and α‐SMA protein levels in extracts from normal BJ fibroblasts (left) and BJ fibroblasts overexpressing hTERT (right) treated with TGF‐β1 for indicated times. (b) Left: Percentage of TIF‐positive BJ fibroblasts that were either control treated (C) or treated with TGF‐β1 for 24 hr in the absence or presence of the Smad3 inhibitor SIS3. Center: quantitation of α‐SMA levels measured by immunoblotting extracts from BJ fibroblasts treated with TGF‐β1 for 24 hr in the absence or presence of the Smad3 inhibitor SIS3. Error bars: ±*SD*. *p < 0.05, (*n* = 3). Representative immunoblot (bottom, γ‐tubulin: loading control) and micrographs (right; α‐SMA: green, 53BP1 red; DAPI: blue) are shown. Scale bars: 500 µm. (c) Same as in B, with the exception that the NOX4 inhibitor VAS2870 (NOX4i) was used instead of SIS3. Error bars: ±*SD*. *p < 0.05, (*n* = 3). (d) Left graph: Percentage of TIF‐positive normal BJ fibroblasts that were either control treated (C) or treated with TGF‐β1 for 24 hr in the absence or presence of the ROS scavenger *N*‐acetyl cysteine (NAC). Error bars: ±*SD*. *p < 0.05, (*n* = 3). Center: quantitation of α‐SMA levels measured by immunoblotting extracts from BJ fibroblasts treated with TGF‐β1 for 24 hr in the absence or presence of the NAC. Error bars: ±*SD*. *p < 0.05, (*n* = 3). Representative immunoblot (bottom, γ‐tubulin: loading control) and micrographs (right; αSMA: green, 53BP1 red; DAPI: blue) are shown. Scale bars: 500 µm. (e) Left graph: Percentage of TIF‐positive normal BJ fibroblasts (−hTERT; left graph) and BJ fibroblasts overexpressing hTERT (+hTERT; right graph) that were either control treated (C), treated with increasing concentrations of H_2_O_2_ for 24 hr, or with zeocin for 4 hr as indicated. Right graph: quantitation of α‐SMA levels measured by immunoblotting extracts from BJ fibroblasts treated with TGF‐β1 for 24 hr in the absence or presence of H_2_O_2_ or zeocin, as indicated. Error bars: ±*SD*. *p < 0.05, (*n* = 3). Representative immunoblot (γ‐tubulin: loading control) and micrographs (right; α‐SMA: green, 53BP1 red; DAPI: blue) are shown. Scale bars: 500 µm

TGF‐β1 stimulates the production of ROS by promoting expression of NADPH oxidase‐4 (NOX‐4) in a SMAD3‐dependent manner (Hecker et al., 2009; Jiang, Liu, Dusting, & Chan, 2014; Sampson et al., 2011). While TGF‐β1‐induced myofibroblast transdifferentiation involves the production of such ROS, the mechanisms how these reactive molecules facilitate transdifferentiation are still obscure. Given that telomeres are sensitive to oxidative stress and rapidly develop aberrant structures, called fragile telomeres, in response to elevated ROS levels we tested the possibility that TGF‐β1 causes telomere dysfunction through SMAD3/NOX4‐dependent ROS production, thereby facilitating transdifferentiation. Consistent with this hypothesis, we demonstrate that addition of TGF‐β1 to the culture medium of BJ fibroblasts caused activation and phosphorylation of Smad3 (Supporting Information Figure S4a) and upregulated the expression of NOX‐4 mRNA and protein in a time‐dependent manner, regardless whether hTERT was expressed or not (Figure 4a). Despite elevated NOX4 levels, however, hTERT‐expressing fibroblasts were resistant to transdifferentiation as demonstrated by the lack of α‐SMA upregulation in these cells (Figure 4a). This demonstrates that hTERT suppresses transdifferentiation downstream of NOX‐4. Significantly, inhibiting SMAD3 or NOX4 activities in normal BJ fibroblasts using pharmacological inhibitors (Supporting Information Figure S4b) not only suppressed formation of dysfunctional telomeres in response to TGF‐β1 treatment, but these inhibitors also prevented myofibroblast transdifferentiation, as evident by the lack of α‐SMA upregulation in these cells (Figure 4b,c). Furthermore, addition of the free radical scavenger *N*‐acetyl cysteine (NAC) to the culture medium of BJ fibroblasts similarly suppressed formation of dysfunctional telomeres and transdifferentiation in response to TGF‐β1 (Figure 4d). Overall, these data demonstrate that active SMAD3/NOX4/ROS signaling promotes both telomere dysfunction and myofibroblast transdifferentiation.

In order to test whether ROS‐induced telomere dysfunction promotes myofibroblast transdifferentiation in the absence of exogenously added TGF‐β1, we incubated BJ fibroblasts and hTERT‐expressing fibroblasts with increasing concentrations of hydrogen peroxide for a period of 24 hr. As demonstrated in Figure 4e, the percentages of TIF‐positive cells that were generated due to ROS were significantly greater in normal BJ fibroblast cultures, compared to BJ‐hTERT cultures. Similarly, while α‐SMA expression levels increased substantially in normal BJ fibroblasts following exposure to ROS, only a modest increase in α‐SMA expression levels was detected in hTERT‐expressing fibroblasts (Figure 4e). In these cells, myofibroblast transdifferentiation could be induced more efficiently using the radiomimetic zeocin, a drug that causes widespread DSB’s (Figure 4e and Supporting Information Figure S4c). This demonstrates that hTERT‐expressing cells resist TGF‐β1‐induced transdifferentiation not because they are insensitive to DDR signaling, but rather because TGF‐β1 fails to induce telomere dysfunction in these cells.

### 2.4 Telomere dysfunction causes myofibroblast transdifferentiation in a p53‐dependent manner

**Figure 5:**
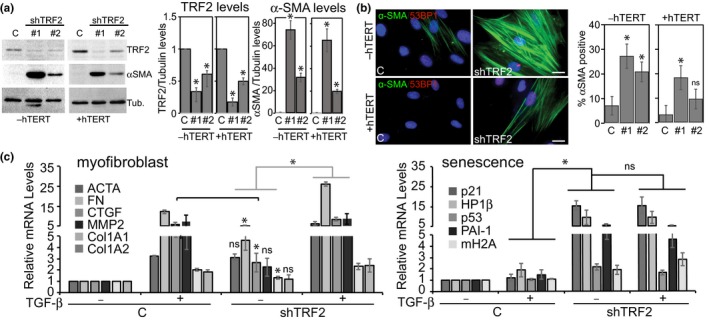
Telomere dysfunction promotes myofibroblast transdifferentiation. (a) Immunoblots of cell extracts from normal BJ fibroblasts (−hTERT; left blot) and BJ fibroblasts overexpressing hTERT (+hTERT; right blot) in which TRF2 had been knocked down using two distinct shRNAs (shTRF2#1, shTRF2#2) or control shRNA (C). γ‐tubulin served as loading control. Right: Fold decrease in TRF2 levels and increase in α‐SMA levels in knockdown cultures as measured by quantitation of the immunoblots. (b) Left: Representative micrographs of control knockdown (left micrographs) and TRF2 knockdown (right micrographs; shTRF2)‐BJ fibroblasts (−hTERT; top) and BJ fibroblasts expressing hTERT (+hTERT; bottom) immunostained using antibodies against α‐SMA (green) and 53PB1 (red). Nuclear DNA was counterstained with DAPI (blue). Scale bars: 20 µm. Right: Quantitation of cells that stained positively for α‐SMA expression in stress fibers, detected using immunofluorescence microscopy, in the indicated TRF2 knockdown cultures of normal BJ fibroblasts (−hTERT) or BJ fibroblasts that overexpress hTERT (+hTERT). Error bars: ±*SD*, *p < 0.02, (*n* = 3), ns: not significant. (c) Left**: **qRT–PCR analysis of indicated myofibroblastic gene expression in control (C) and TRF2 knockdown cultures in the presence or absence of TGF‐β1 treatment for 48 hr. Error bars: ±SE (*n* = 3). *p < 0.005 as determined by two‐way ANNOVA analysis; ns: not significant. Right: qRT–PCR analysis of indicated senescence gene expression in control and TRF2 knockdown cultures in the presence or absence of TGB‐β1 treatment for 48 hr. Error bars: ±*SE* (*n* = 3) *p < 0.005 as determined by two‐way ANOVA analysis. ns: not significant. Cells were analyzed 12–16 days following retroviral transductions of TRF2 shRNA’s

Our data suggest that ROS‐induced telomere dysfunction causes fibroblasts to myofibroblasts transdifferentiation. It is possible, however, that ROS activate additional pathways that contribute to myofibroblast transdifferentiation, pathways that potentially are critical for this process. To directly test whether telomere dysfunction is sufficient for myofibroblast transdifferentiation, we suppressed the expression of TRF2, a shelterin component that is essential for telomere capping and function (Palm & de Lange, 2008). Using two distinct shRNA’s, we stably knocked down TRF2 protein expression to different levels, which resulted in rapid telomere dysfunction in both normal BJ fibroblasts and, to a lesser degree, also in fibroblasts overexpressing hTERT (Figure 5a and Supporting Information Figure S5a), which is consistent with previous studies (Takai et al., 2003). Strikingly, in both normal and hTERT‐expressing fibroblasts, we observed a dramatic upregulation of α‐SMA to levels that were equivalent to the efficiency of TRF2 knockdown (Figure 5a). In addition, TRF2 knockdown resulted in a significant increase in cells that expressed α‐SMA in stress fibers (Figure 5b), suggesting that telomere dysfunction causes myofibroblast transdifferentiation, even in the absence of exogenously added TGF‐β1 and ROS. Some cells that expressed α‐SMA in stress fibers also expressed high levels of macroH2A and compared to TGF‐β1‐treated cells, TRF2 knockdown resulted in a greater abundance of cells positive for SA‐βGal activity and reduced levels of lamin B1 (Supporting Information Figure S5b–d), features of senescent human fibroblasts. To test the relationship between telomere dysfunction‐induced senescence and telomere dysfunction‐induced transdifferentiation in greater detail, we compared gene expression profiles that characterize myofibroblast and senescent cells following TGF‐β1 treatment, TRF2 knockdown, or both. While knockdown of TRF2 upregulated expression of α‐SMA to a similar degree compared to TGF‐β1‐treated cells, expression of other myofibroblast genes was generally less efficient in TRF2 knockdown cells (Figure 5c). Similarly, compared to TRF2 knockdown that efficiently caused upregulation of genes associated with the senescence response, TGF‐β1 only minimally upregulated expression of senescence genes (Figure 5c). However, additionally stimulating TRF2 knockdown cells with TGF‐β1 was able to increase expression of myofibroblast genes to levels similar or greater compared to TGF‐β1 only treated cells, but it did not further increase expression of senescence genes (Figure 5c). Our data therefore suggest that telomere dysfunction is necessary, but not sufficient for fibroblast to myofibroblast transdifferentiation.

**Figure 6:**
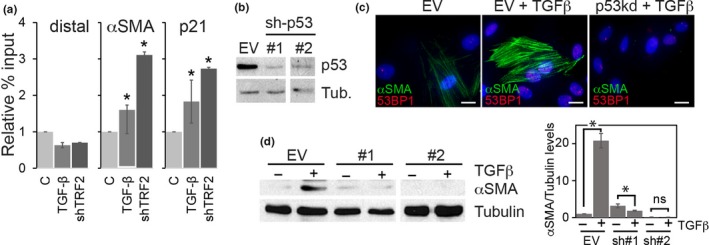
Telomere dysfunction causes myofibroblast transdifferentiation in a p53‐dependent manner. (a) ChIP‐qPCR analysis of p53 binding to a control distal promoter element (distal), to the αSMA promoter element (αSMA), or the p21 promoter element (p21) of normal BJ fibroblasts that were either control treated (C), treated with TGF‐β1 (10 ng/ml) for 48 hr, or transduced with shRNA targeting TRF2. Error bars: ±*SD*. *p < 0.05, (*n* = 3). (b) p53 immunoblot of BJ fibroblast cell extracts in which expression of p53 had been knocked down using two distinct shRNAs (#1 and #2). EV: knockdown using control empty vector. (c) Representative micrographs of untreated control knockdown cultures (EV; left) and TGF‐β1 treated (10 ng/ml; 48 hr) cultures in which p53 had been knocked down using shRNA (p53kd; right) or control (EV; center) that were immunostained using antibodies against α‐SMA (green) and 53PB1 (red). Nuclear DNA was counterstained with DAPI (blue). Scale bars: 20 m. (d) α‐SMA immunoblot of BJ fibroblast whole cell extracts expressing two distinct shRNAs targeting p53 (#1–2) or empty vector control (EV) that were either control treated (−) or treated with TGF‐β1 for 48 hr (+). γ‐tubulin was used as loading control. Bar graph: fold differences in α‐SMA expression levels quantified from immunoblots. ±*SD*. *p < 0.05, (*n* = 3)

α‐SMA is a direct transcriptional target of the tumor suppressor p53 and activation of a temperature sensitive p53 allele in rat embryo fibroblasts causes these cells to upregulate and express α‐SMA in actin filament bundles that resemble stress fibers of myofibroblasts (Comer et al., 1998). Since telomere dysfunction activates a p53 dependent DNA damage checkpoint (d’Adda di Fagagna et al., 2003; Herbig et al., 2004; Takai et al., 2003), it is possible that TGFβ1‐induced telomere dysfunction contributes to α‐SMA upregulation and transdifferentiation of myofibroblasts by promoting p53‐dependent α‐SMA expression. Consistent with this interpretation, we demonstrate, using ChIP, that p53 binds to the α‐SMA promoter in BJ fibroblasts following TGF‐β1‐treatment (Figure 6a). Similarly, inducing telomere dysfunction by shRNA mediated knockdown of TRF2 also caused p53 to bind to the α‐SMA promoter, suggesting that telomere dysfunction‐induced p53 activation contributes to myofibroblast transdifferentiation in humans. To directly assess whether telomere dysfunction‐induced transdifferentiation is dependent on p53, we suppressed p53 expression levels in BJ fibroblasts using two distinct shRNAs (Figure 6b) and monitored the ability of fibroblasts to transdifferentiate into myofibroblasts following stimulation with TGF‐β1. While control knockdown cultures upregulated α‐SMA expression in response to TGF‐β1 treatment, cultures in which p53 had been knocked down were essentially blocked from transdifferentiation, as demonstrated by the absence of α‐SMA expression following TGF‐β1 treatment (Figure 6c,d). Similar results were observed in BJ fibroblasts in which p53 activity was blocked due to retroviral expression of SV40 large T antigen or HPV E6 (Supporting Information Figure S6). Our data therefore demonstrate that TGFβ1‐induced telomere dysfunction promotes myofibroblast transdifferentiation by facilitating p53‐dependent transactivation of α‐SMA expression.

## 3 DISCUSSION

TDIS has been demonstrated to function as a tumor‐suppressing mechanism in mice (Cosme‐Blanco et al., 2007; Feldser & Greider, 2007) and in humans (Suram et al., 2012), where it arrests proliferation of epithelial and melanocytic cells in early neoplastic lesions. Telomere dysfunction, therefore, forces cells that are at risk for malignant transformation to abruptly cease proliferation and lose function. The data presented here now reveal that telomere dysfunction also promotes a gain‐of‐function phenotype, as it mediates the events that cause transdifferentiation of fibroblasts into myofibroblasts. Our data thus demonstrate that, depending on cell type, telomere dysfunction can either initiate a mechanism that causes cells to cease proliferation and lose function or, alternatively, activate a program that causes cells to differentiate into a cell type with designated function.

After tissue damage, the transient generation of senescent stromal cells at the site of injury is critical to suppress fibrosis (Jun & Lau, 2010; Krizhanovsky et al., 2008), attract immune modulatory cells (Krizhanovsky et al., 2008), and promote the generation of myofibroblasts in a paracrine manner (Demaria et al., 2014). While the formation of myofibroblasts during wound healing in a mouse model system was reported to be stimulated by SASP factors secreted from senescent fibroblasts and endothelial cells generated at the site of injury (Demaria et al., 2014), other studies demonstrated that, paradoxically, the SASP causes bystander senescence in somatic human fibroblasts (Acosta et al., 2013; Hubackova et al., 2012; Nelson et al., 2012). Recent studies, however, have raised the possibility that the SASP can promote development of myofibroblasts, as fibroblast incubated with conditioned medium from senescent cells develop into contractile α‐SMA‐expressing cells (Schafer et al., 2017). Although it is possible that different senescence‐inducing signals activate the production of distinct senescence‐associated secretomes that may either trigger a transdifferentiation program or alternatively, bystander senescence in human fibroblasts, our results demonstrate that these two events are similar.

In this study, we demonstrate that TGF‐β1, a major component of the early SASP, promotes myofibroblasts transdifferentiation in a manner that is dependent on telomere dysfunction, a strong senescence‐inducing signal. The observations that a DDR induced by radiation, drugs, or H2O2 similarly promotes myofibroblast transdifferentiation support this conclusion. (Dimitrijevic‐Bussod et al., 1999; Mellone et al., 2016; Sampson et al., 2011). In fact, myofibroblasts have been reported to be nonproliferating cells that show signs of DNA damage (Petrov et al., 2008). Significantly, our study reveals that this DNA damage is due to telomere dysfunction. Furthermore, data presented here also reconcile seemingly contradictory observations that TGF‐β1, the primary factor that activates fibroblasts to myofibroblast transdifferentiation in cell cultures and during wound healing (Hinz, 2007), can cause paracrine senescence in human fibroblasts (Hubackova et al., 2012). As our data demonstrate that early passage human fibroblasts with dysfunctional telomeres transdifferentiate into functional myofibroblasts while displaying some features of senescence, they suggest that functional myofibroblasts and senescent (myo)fibroblasts are difficult to distinguish. The observations that fibroblast with senescence features detected in damaged tissue frequently display features of myofibroblasts, such as high expression levels of α‐SMA, are consistent with this conclusion (Jun & Lau, 2010) (Demaria et al., 2014; Krizhanovsky et al., 2008). It is important to note, however, that human fibroblasts serially passaged into replicative senescence and those that underwent oncogene‐induced senescence, both lacked features of myofibroblasts, demonstrating that not all fibroblasts induced to senesce by telomere dysfunction or other stresses are functional myofibroblasts. It remains to be determined why senescent cells are resistant to myofibroblast transdifferentiation in response to TGF‐β1 or telomere dysfunction.

TGF‐β1 signaling can either promote cellular proliferation or suppress it depending on the genetic context of the cell (Mueller & Fusenig, 2004). Since ROS, generated through the TGF‐β1/Smad3/NOX4‐signaling pathway, caused telomere dysfunction and proliferative arrest of human somatic fibroblasts that lack detectable telomerase activity, but did not affect proliferation rates of fibroblasts expressing hTERT, our data suggest that opposing effects of TGF‐β1 on cell proliferation depend on the levels of telomerase activity in a cell. The insensitivity of hTERT‐expressing fibroblasts to TGF‐β1‐induced growth arrest and myofibroblast transdifferentiation likely is due to the ability to telomerase to suppress the formation of dysfunctional telomeres in cells encountering ROS‐induced telomeric replication stress, as demonstrated previously (Boccardi et al., 2015). As hTERT expression reduced formation of both telomeric and to some extent also nontelomeric DDR foci, it is also possible, however, that noncanonical functions of telomerase, such as its ability to reduce cellular ROS production (Saretzki, 2014), contribute to suppressing activation of a DDR and myofibroblast transdifferentiation of human fibroblasts.

In concordance with our conclusions, it was previously shown that inhibiting telomerase activity or suppressing expression of hTERT caused fibroblast to transdifferentiate into myofibroblasts (Liu et al., 2006). Since telomerase activity is transiently elevated for several days in fibroblasts following tissue damage (Minamino, Mitsialis, & Kourembanas, 2001; Nozaki, Liu, Hatano, Gharaee‐Kermani, & Phan, 2000; Tahara, Sato, Noda, & Ide, 1995), it was suggested that it promotes fibroblast proliferation while antagonizing myofibroblast transdifferentiation during the proliferative stage of tissue repair (Schissel & Layne, 2006). A decrease in telomerase activity is observed several weeks following tissue damage and during a period in which abundance of myofibroblasts increases (Liu et al., 2006; Liu, Nozaki, & Phan, 2002; Nozaki et al., 2000), raising the possibility that temporally emerging signals in damaged tissue eventually repress hTERT expression, thereby facilitating myofibroblast transdifferentiation. The likely factor that orchestrates all of the events critical for myofibroblast transdifferentiation during tissue repair and wound healing in humans is TGF‐β1, as it negatively regulates transactivation of hTERT expression (Cassar et al., 2016), promotes telomere dysfunction and p53‐dependent expression of αSMA (this study), and activates SMAD3‐dependent expression of αSMA (Hinz, 2007).

Although a direct involvement of p53 in α‐SMA transactivation or myofibroblasts transdifferentiation has not been reported, to the best of our knowledge, it has been established that p53 cooperates with Smad transcription factors in the activation of a number of TGF‐β1 target genes (Dupont et al., 2004). Since both, Smad3 (Hinz, 2007) and p53 (this study), are required for TGF‐β1‐induced expression of αSMA, a factor critical for myofibroblast function, this cooperation likely also promotes αSMA expression during transdifferentiation. While our data reveal a novel and essential role for telomere dysfunction and p53 in transdifferentiation of human fibroblasts, it remains to be determined whether telomere dysfunction and its associated p53‐dependent DDR are involved also during differentiation of other human cell types.

## 4 EXPERIMENTAL PROCEDURES

### 4.1 Cell culture and reagents

Human foreskin fibroblasts, BJ cells (ATCC), GM21‐human foreskin fibroblasts (Coriell, NJ), LF1‐human lung fibroblasts and WI38‐human lung fibroblasts and derivatives were cultured in Ham’s F10 nutrient mixture (Life Technologies) supplemented with 10% batch‐tested fetal bovine serum (Atlanta Biologicals; Lawrenceville, GA). Cultures were passaged at 1:4 in 10 cm dishes (Corning) and incubated at 37°C in atmosphere of 5% CO_2__%_ and 2% Oxygen. To prepare conditioned medium (CM), cells were washed three times with serum free medium followed by a 48 hr incubation in 10 ml serum free medium. Following CM collection, cells were counted to determine input volume for CM. Floating debris was removed from CM filtration using a 0.45 µm pore filter. CM was either used immediately or stored at −80°C until further use. Prior to use, CM was supplemented with 10% FBS. BJ‐hTERT cells were generated by retroviral transduction of BJ cells using the pBabe‐hTERT‐puro vector followed by drug selection. Cells were treated with 10 ng/ml recombinant TGF‐β1 (R&D systems) in medium containing 10% FBS, unless otherwise indicated. Hydroxyurea (Sigma, St Louis, MO; 50 μM), H_2_O_2_ (Fisher Scientific; 50 and 100 μM), and zeocin (Millipore; 4 μg/ml) were directly added to the culture medium. Cell proliferation curves were generated by plating a fixed number of cells and counting the number of cells recovered from the dish each day using a hemocytometer. TGF‐β receptor 1 inhibitor SB431542, phospho‐Smad3 inhibitor SIS3, and Nox‐4 inhibitor VAS2870 were all procured from Sigma‐Aldrich (St Louis, MO) and preincubated with cells for one hour before the addition of TGF‐β1.

### 4.2 Cell proliferation assay

Cell proliferation curves were generated by plating a fixed number of cells and counting the number of cells recovered from the dish each day using a hemocytometer. To measure EdU incorporation, proliferating cells were labeled with 10 μM EdU for 12 hr, and EdU‐positive cells were detected by using the ClickiT EdU Alexa Fluor 488 imaging kit (Invitrogen) according to manufacturer’s instructions.

### 4.3 Viral transductions

BJ and BJ‐hTERT cells were transduced with lentiviral particles encoding shRNAs against TRF2 and p53 (Sigma‐Aldrich, St Louis, MO). BJ cells expressing SV40 Large T antigen (Addgene; #1780) and HPV E6 cells were generated by retroviral transductions with pBabe‐Neo‐SV40 Large T antigen plasmid and pBabe‐puro‐HPV‐E6 plasmid, respectively. GM21 cells expressing inducible hTERT were described previously (Patel et al., 2016). To generate fibroblasts in oncogene‐induced senescence, BJ cells were transduced with pBABE‐Puro H‐Ras^G12V^ (Addgene; #1768), selected with puromycin for 48 hr and allowing cells to undergo senescence for 14 days. Retroviral and lentiviral particles were generated by calcium phosphate transfection of Plat‐A and Phoenix amphotropic virus packaging cell lines. After transfection, viral supernatants containing high titer retrovirus (viral particles that cause a transduction efficiency of at least 70% of cells) were collected and incubated with ~65% confluent BJ cells for 8–12 hr. Cells were selected with 2 µg/ml puromycin (Sigma‐Aldrich, St Louis, MO) for 48 hr or 500µ g/ml of neomycin for 7 days.

### 4.4 ImmunoFISH and immunofluorescence microscopy

Cultured cells were processed for immunofluorescence analysis as described previously (Herbig et al., 2004). Primary antibodies were incubated overnight at 4°C in block buffer (4% BSA in PBST) at indicated concentrations (see below). Following 2 × 5 min washes with PBST, cells were incubated with secondary antibodies as indicated (1:1,000 in block buffer) for 1 hr at room temperature. Cells were washed 3 × 5 min with PBS, rinsed with water, and mounted using DAPI containing mounting medium (Vector Laboratories, Burlingame, CA). To detect TIF, fixed cells were dehydrated by sequentially placing them in 70% EtOH, 90% EtOH, and 100% EtOH for 3 min each. After air‐drying, nuclear DNA was denatured for 5 min at 80°C in hybridization buffer containing Cy3‐conjugated telomere‐specific peptide nucleic acid (PNA; Cy3‐(C_3_TA_2_)_3_; Panagene, Korea) at 0.5 μg/ml, 70% formamide, 12 mM Tris‐HCl pH = 8.0, 5 mM KCl, 1 mM MgCl_2_, 0.08% Triton X‐100%, and 0.25% acetylated BSA (Sigma‐Aldrich, St Louis, MO), followed by incubation in the same buffer for 2 hr at room temperature. Slides were washed sequentially with 70% formamide/0.6× SSC (90 mM NaCl, 9 mM Na‐citrate [pH = 7]; 3 × 15 min), 2× SSC (15 min), PBS (5 min), PBST (5 min), and incubated with block buffer (4% BSA in PBST) for 30 min. Immunostaining using primary polyclonal anti‐53BP1 antibodies and secondary AlexaFluor 488‐conjugated goat anti‐rabbit antibodies (Invitrogen, Carlsbad, CA) was performed as described above. Cells were mounted and analyzed by immunofluorescence microscopy using a Zeiss Axiovert 200 fluorescence microscope, an AxioCamMRm camera (Zeiss), and AxioVision 4.6.3 software (Zeiss). To analyze and quantitate colocalizations between telomere signals and 53BP1 foci, images were acquired as z‐stacks using a 100/1.4 oil immersion lens, and an ApoTome (Zeiss). ApoTome microscopy eliminates out of focus light and generates shallow focal planes (0.4 µm using a 100× oil objective). Stacks were merged into a single image using the AxioVision software for easier counting. A cell was considered as TIF positive when 50% or greater of the nuclear DDR foci colocalized with telomeric repeats. Relative telomere lengths were assessed by quantification of telomeric signal fluorescence intensities using the ImageJ software (version 1.45) and the object counter 3D plugin on consecutive z‐stacks.

### 4.5 Senescence‐associated β‐galactosidase activity

Cells were washed and fixed with 2% formaldehyde +0.2% glutaraldehyde in PBS, for 3–5 min, washed again and incubated at 37°C overnight in staining solution (40 mM citric acid/sodium phosphate solution, pH 6.0, 1% 5‐bromo‐4‐chloro‐3‐indolyl‐β‐D‐galactopyranoside (X‐gal), 5 mM potassium ferrocyanide, 5 mM ferricyanide, 150 mM sodium chloride, and 2 mM magnesium chloride) as per (Dimri et. al., 1995).

### 4.6 Antibodies

The sources and dilutions of antibodies used were as follows: α‐SMA (SIGMA‐Aldrich, 1:1,000), 53BP1 (polyclonal; Novus, Littleton, CO), β‐Tubulin, p53(S15), phospho‐SMAD3 (Cell Signaling, Danvers, MA); p‐ATM(S1981) (Abcam, Cambridge, MA); p21 (Santa Cruz, Dallas, TX), TRF2 (Novus, Littleton, CO; 1:500), p16 (Santa Cruz, CA; 1:200); Nox‐4 (Abcam, Cambridge, MA); macroH2A (kind gift from Dr. Peter Adams), Lamin B1 (Abcam; 1:1,000); Ras (BD Transduction Laboratories, San Jose, CA; 1:1,000); p53 (DO‐1, BD biosciences); γH2AX(S139) (Upstate, Chicago, IL; 1:1,000); γ‐Tubulin (Sigma, St Louis, MO; 1:5,000).

### 4.7 Protein extraction and immunoblotting

Protein extracts were prepared in RIPA lysis buffer (20 mM Hepes‐KOH, pH7.9, 0.42 M KCL, 25% glycerol, 0.1 mM EDTA, 5 mM MgCl2, 0.2% NP40, 1 mM DTT, 500 μM PMSF, and 1:100 protease inhibitor cocktail (Sigma‐Aldrich, St Louis, MO). 30 µg protein samples were run on 12% SDS‐PAGE gels and proteins were transferred to PVDF membranes (Pall Life Sciences; Pensacola, FL) using a Bio‐Rad mini Trans‐Blot Cell at 350 mA for 90 min. After transfer, membranes were blocked in 5% nonfat dry milk in 1× TBST (150 mM NaCl, 10 mM Tris‐HCl, pH 8.0, 0.05% Tween 20) at room temperature for 1 hr, then incubated with primary antibody at 4°C overnight with gentle agitation. Membranes were washed three times in 1× TBST for 10 min and subsequently incubated with HRP‐conjugated goat anti‐mouse or goat anti‐rabbit secondary antibodies (Pierce Biotechnology; Rockford, IL) at room temperature for 1 hr with shaking. Membranes were washed three times in 1× TBST for 10 min. Proteins were detected with a SuperSignal West Pico Chemiluminescent Substrate (Pierce Biotechnology; Rockford, IL) and signals were exposed to Hyblot CL Autoradiography films (Denville; Metuchen, NJ). Autoradiography films were scanned and band signal intensities were measured by densitometry using the Image J software (version 1.45). γ‐tubulin or ponceau S stain was used as a loading control as indicated.

### 4.8 Quantitative reverse transcription–PCR

RNA was isolated from cells with the Qiagen RNAeasy kit according to the manufacturer’s instructions. One microgram of total RNA was subjected to reverse transcription with random hexamer primers using the Bio‐Rad Advanced cDNA Synthesis Kit according to the manufacturer’s instructions (Bio‐Rad, Hercules, CA). Quantitative PCR was performed using Bio‐Rad Real Time PCR detection system with the SYBR Green PCR Master Mix (Bio‐Rad). The following primers were used: **COL1A1** forward (5′‐CACACGTCTCGGTCATGGTA‐3′) and reverse (5′‐CGGCTCCTGCTCCTCTTAG‐3′); **COL1A2** forward (5′‐AGCAGGTCCTTGGAAACCTT‐3′) and reverse (5′‐GAAAAGGAGTTGGACTTGGC‐3′); **ACTA2** (α‐SMA) forward (5′‐GATGGCCACTGCCGCATCCT‐3′) and reverse (5′‐ACAGGGTCTCTGGGCAGCGG‐3′); **MMP9** forward (5′ GGTGATTGACGACGCCTTTGC 3′) and reverse (5′ CGCGACACCAAACTGGATGAC 3′); **TIMP** forward (5′ CCAGGACGCCTTCTGCAAC 3′) and reverse (5′ CCTCCTTTACCAGCTTCTTCCC 3′); **Fibronectin** forward (5′‐CCA TCG CAA ACC GCT GCC AT‐3′) and reverse (5′‐AAC ACT TCT CAG CTA TGG GCT T‐3′); **GAPDH** forward (5′‐AAG AAG GTG GTG AAG CAG GC‐3′) and reverse (5′‐TCC ACC ACC CTG TTG CTG TA‐3′); **CTGF** forward (5′‐CAGGCTGGGGAGAAGCAGAGTCGT‐3′) and reverse (5′‐CTGGTGCAGCCAGAAAGCTCAA‐3′); **macroH2A** forward (5′‐ AACAAGAAGGCCCGGATAGC‐3′) and reverse (5′‐CCTTTTAGCAGCTGGTTGAGC); **p53** forward (5′‐ TTCCGAGAGCTGAATGAGGC‐3′) and reverse (5′‐CTTCAGGTGGCTGGAGTGAG‐3′); CDKN1A (Qiagen Quantitect primer Assay, Cat # QT00062090); **MMP2** forward (GGCAACATGACCAGCTG) and reverse (CAAGGTGCTGGCTGAGTAGATC); **PAI‐1 **(Plasminogen Activator Inhibitor) Forward (GGCAACATGACCAGCTG) and reverse (GGCCAAGTGATGGAACCC); **HP1‐β** forward (5′‐ CTTTGCAGGACTACGGAGGAG‐3′) and reverse (5′‐ GTGTAAAGGGTGACGCTGCTTG‐3′); Relative changes in mRNA expression were calculated using GAPDH as an internal reference.

### 4.9 Fibroblast populated collagen lattice assay

To assess contractile abilities, collagen contraction assays were carried out using BJ and BJ‐hTERT fibroblasts based on Tomasek and Rayan (Tomasek & Rayan, 1995). Collagen lattices were cast in 24‐well tissue culture plates. For each well, 1 × 10^5^ cells were quickly mixed with 400 μl of type I, rat tail collagen (final concentration of 1.8 mg/ml), 100 μl of neutralization solution (three parts waymouth: two parts 0.34 M NaOH), and 2 μg/ml human recombinant TGF‐β1 or solvent. Collagen lattices were incubated at 37°C in 5% CO_2_ for 1 hr to allow for collagen polymerization. Wells were then flooded with 1 ml of Hams F‐10 supplemented with 10% FBS. The collagen cultures were maintained for 48 hr. During this time, the primary fibroblasts in three‐dimensional cultures respond to stress within the tethered, polymerized collagen lattice and differentiate into contractile myofibroblasts. After two days in culture, collagen lattices were simultaneously released from the wells using a metal spatula, thereby allowing the differentiated myofibroblasts to contract the untethered lattice. Floating lattices were digitally scanned at 0, 24, 48, and 96 hr after release, and the areas of each lattice were determined using the freehand area tool in Axiovision software. Sequential area calculations were normalized to the area of the well prior to release and expressed as % contraction. At each time point, the area of the lattice was normalized to the cell number at that time point in order to account for the difference between the proliferative rates of BJ and BJ‐hTERT cells.

### 4.10 Chromatin Immunoprecipitation assay

Chromatin Immunoprecipitation (ChIP) was carried out according to the manufacturer’s instructions (EMD Millipore, Billerica, MA). Cells were cross‐linked by incubating them in 1% (vol/vol) formaldehyde containing medium for 10 min at 37°C and then sonicated 6 times for 5 s each to obtain DNA fragments 200–1,000 bp in length. Antibodies against human p53 (BD biosciences DO‐1) and phospho‐Smad3 were used to precipitate DNA fragments and the protein‐DNA complex was collected with protein‐G sepharose beads, eluted and reverse cross‐linked. Extracted samples were then purified using spin columns and recovered DNA was used for quantitative RT–PCR. Primer sequences for the ACTA promoter were forward primer 5′‐ATTCCTATTTCCACTCAC‐3′and reverse primer 5′‐ACTTGCTTCCCAAACA‐3′). Primers for the p53‐binding motif were 5′ GCCCCTTTCTGTTCTCAGTT 3′ (forward) and 5′ TGTGGGAGATAAACACGCCA 3′ (reverse).

## CONFLICT OF INTEREST

The authors declare that no conflict of interest exists.

## AUTHOR CONTRIBUTIONS

NR performed all experiments In Figures 1, 2, 3, 4, 5, 6 and Supporting Information Figures S1, S2, S3d,e, S3g‐i, S3k, S4, S5 analyzed the data and assembled images. TV performed experiments in Supporting Information Figures S3a–c, S3f, S3j‐k, S6. UH and NR designed the study and wrote the manuscript.
