# Supplementary material for: Telomere dysfunction promotes transdifferentiation of human fibroblasts into myofibroblasts
Source: Aging Cell. 2018 Sep 22;17(6):e12838. doi: 10.1111/acel.12838 (PMC6260909; doi:10.1111/acel.12838)
Supplement: Supplementary file 1 [file ACEL-17-e12838-s001.docx]

**Supplementary Figures**


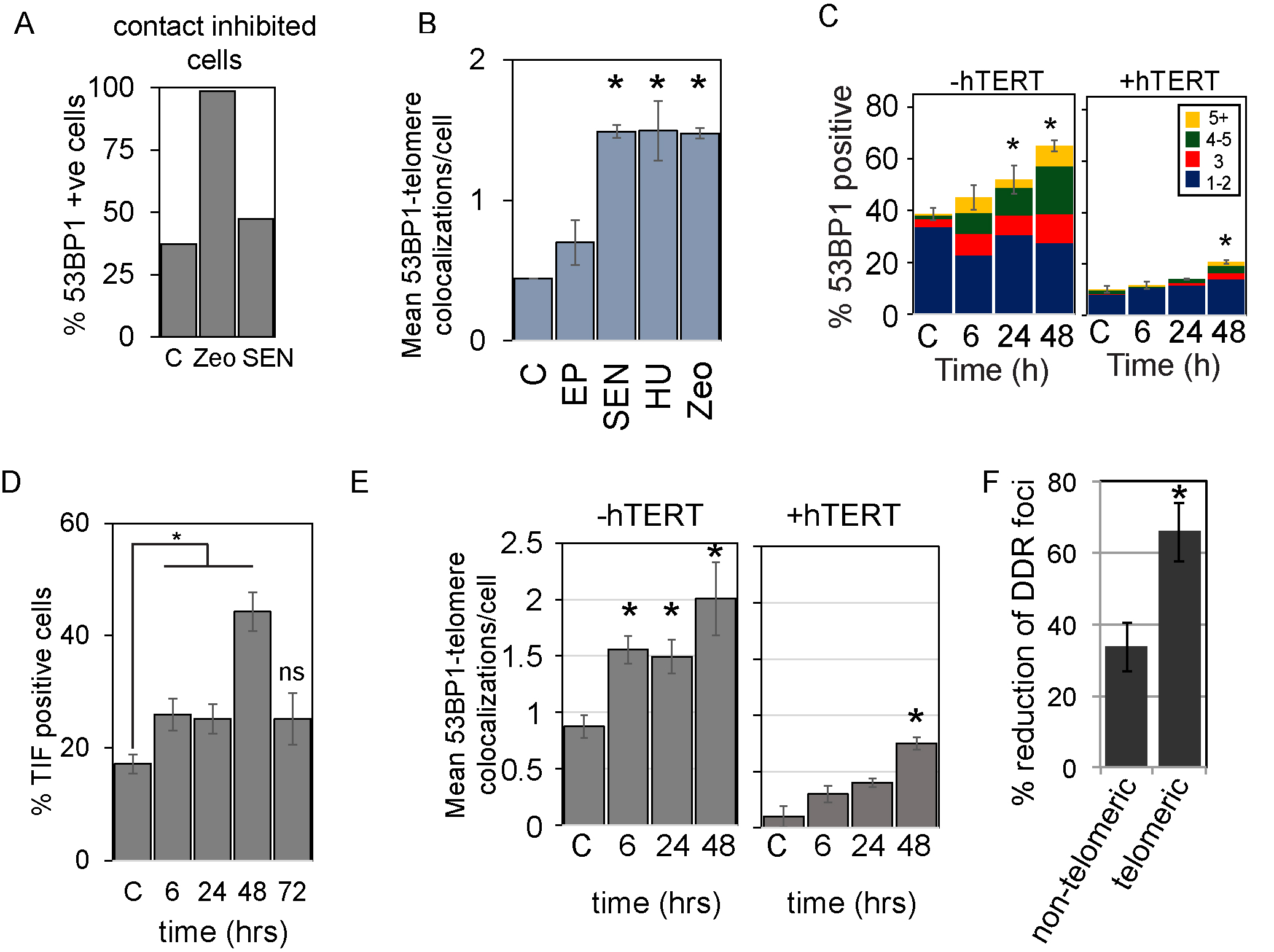


**Figure S1**. **Fibroblasts, but not hTERT expressing fibroblasts, rapidly develop DDR foci and dysfunctional telomeres in response to the SASP. A** Percentage of contact inhibited BJ fibroblasts that developed at least one 53BP1 focus following a 48h incubation with conditioned medium from replicatively senescent cells (SEN). Untreated early passage cells (C) and zeocin treated cells (Zeo) are also shown as controls **B** Mean 53BP1-telomere colocalizations in fibroblasts that were untreated (C), or incubated with conditioned medium from early passage cells (EP), replicatively senescent cells (SEN), hydroxyurea treated cells (HU), and zeocin treated cells (Zeo), and for 48h. Error bars: +/-SD. *: p<0.05 (n=3) **C** Percentage of normal human BJ fibroblasts (-hTERT; left graph) and BJ fibroblasts overexpressing hTERT (+hTERT; right graph) positive for indicated number of 53BP1 foci after treatment with senescent cell-conditioned medium for indicated times. C: control untreated cells. Error bars: +/-SD. *: p<0.05 (n=3) **D** Percentage of normal human BJ fibroblasts positive for TIF after treatment with senescent cell-conditioned medium for indicated times. C: control untreated cells. Error bars: +/-SD. *: p<0.05 (n=3) **E** Mean 53BP1-telomere colocalizations in normal human BJ fibroblasts (-hTERT; left graph) and BJ fibroblasts overexpressing hTERT (+hTERT; right graph) after treatment with senescent cell-conditioned medium for indicated times. C: control untreated cells. Error bars: +/-SD. *: p<0.05 (n=3). **F** Percent reduction of non-telomeric and telomeric DDR foci in hTERT expressing BJ fibroblasts, compared to normal BJ fibroblasts. Error bars: +/-SD. *: p<0.05

**
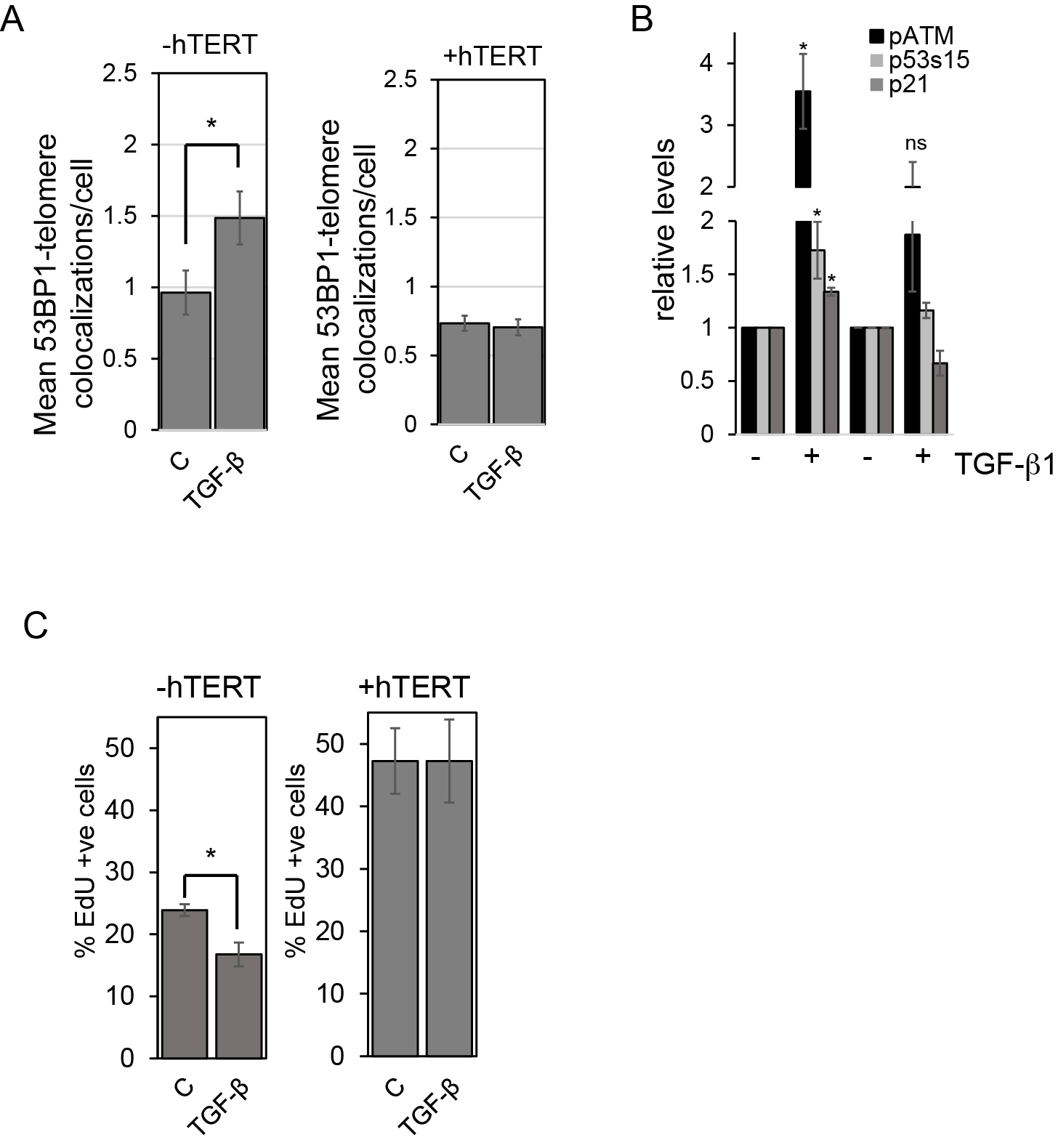
**

**Figure S2. TGF-β1 causes telomeric DDR foci and proliferative defects in fibroblasts that lack hTERT expression A** Mean 53BP1-telomere colocalizations in normal BJ fibroblasts (-hTERT) or BJ fibroblasts overexpressing hTERT (+hTERT) that were untreated (C), or incubated with TGF-β1 (10ng/ml) for 48h. Error bars: +/-SD. *: p<0.05 (n=3). **B** Quantitation of immunoblots shown in Figure 2E. Error bars: +/-SD. *: p<0.05 (n=3). **C** Percentages of BJ fibroblasts (-hTERT) or BJ fibroblasts overexpressing hTERT (+hTERT) treated as in A that incorporated EdU for 12h. Error bars: +/-SD. *: p<0.05 (n=3).

**
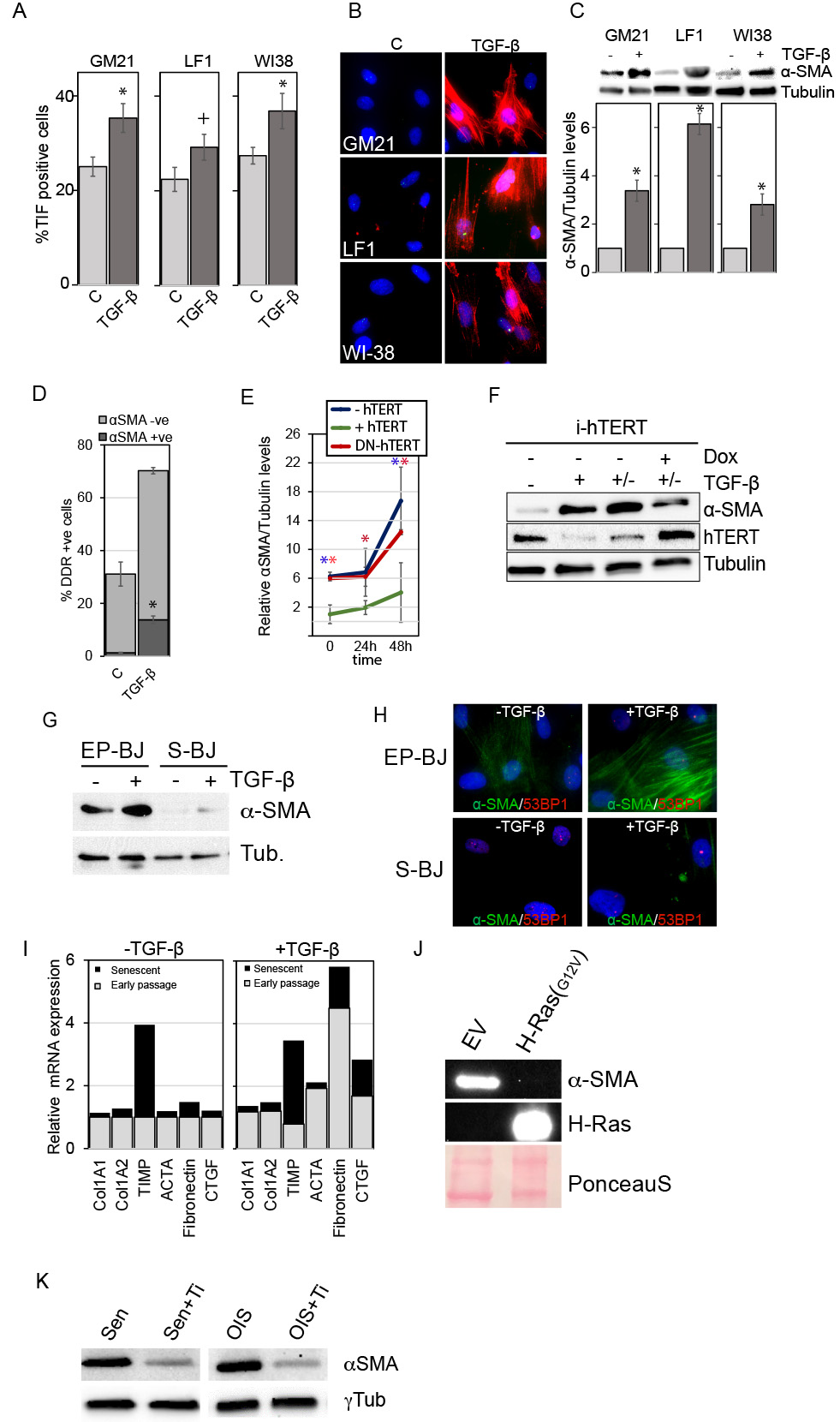
**

**Figure S3. Normal human fibroblasts, but not senescent fibroblasts, develop dysfunctional telomeres and features of myofibroblasts following TGF-β1 treatment. A** Percentage of TIF positive GM21, LF1, and Wi38 human fibroblasts following control treatment (C) or 48h incubation with TGF-β1 (10ng/ml). Error bars: +/-SD. *: p<0.05, +: 0.07 (n=3) **B** Representative micrographs of indicated fibroblast strains immunostained using antibodies against 53BP1 (green) and α-SMA (red) that were either untreated (left column) or treated with TGF-β1 (right column) as in A.. Cell nuclei were counterstained with DAPI (blue). **C** Cell extracts from indicated human fibroblasts strains and treated as in A were immunoblotted using antibodies against αSMA and γ-Tubulin as loading control. Bar graphs: quantitation of αSMA levels from immunoblots. Error bars: +/-SD. *: p<0.05 (n=3). **D** Percentages of 53BP1 positive BJ fibroblasts (DDR positive) that were also positive (dark grey bars) or negative (light grey bars) for α-SMA expression in stress fibers following control (C) and TGF-β1 (10 ng/ml) treatment for 48 h. Error bars: +/-SD. *: p<0.05 (n=3). **E** Quantitation of α-SMA expression levels from immunoblots as in Figure 3D. Error bars: +/-SD. *: p<0.05 (n=3). **F** Immunoblot using antibodies against indicated proteins of GM21 human fibroblasts expressing hTERT from a doxycycline inducible promoter were either untreated (-) or treated with 10ng/ml TGF-β1 for 48h (+), followed by a 48h recovery period in the absence of TGF-β1 (+/-). + Dox: doxycycline was added for 48h during the recovery period. **G** Immunoblot using indicated antibodies of early passage (EP-BJ) and senescent BJ fibroblasts (S-BJ) that were either untreated (-) or treated (+) with TGF-β1 (10 ng/ml) for 48 h. **H** Immunofluorescence analysis using antibodies against α-SMA (green) and 53BP1 (red) of fibroblasts as in G. DAPI: blue. **I** qRT-PCR analysis of characteristic myofibroblastic genes in the absence of TGB-β treatment (left graph) and following TGF-β treatment for 48h (right graph) in early passage (light grey bars) and senescent BJ fibroblasts (dark grey bars). **J** Immunoblot using indicated antibodies in H-RasG12V or empty vector (EV) retrovirally transduced human fibroblasts, 23 days following transduction. Loading control: Ponceau S stain. **K** Immunoblots using indicated antibodies of early passage BJ fibroblasts treated for 48h with conditioned medium from replicatively senescent (sen) and oncogene (H-RasG12V)-induced senescent cells in the absence or presence of a TGF-β1 receptor inhibitor (Ti).


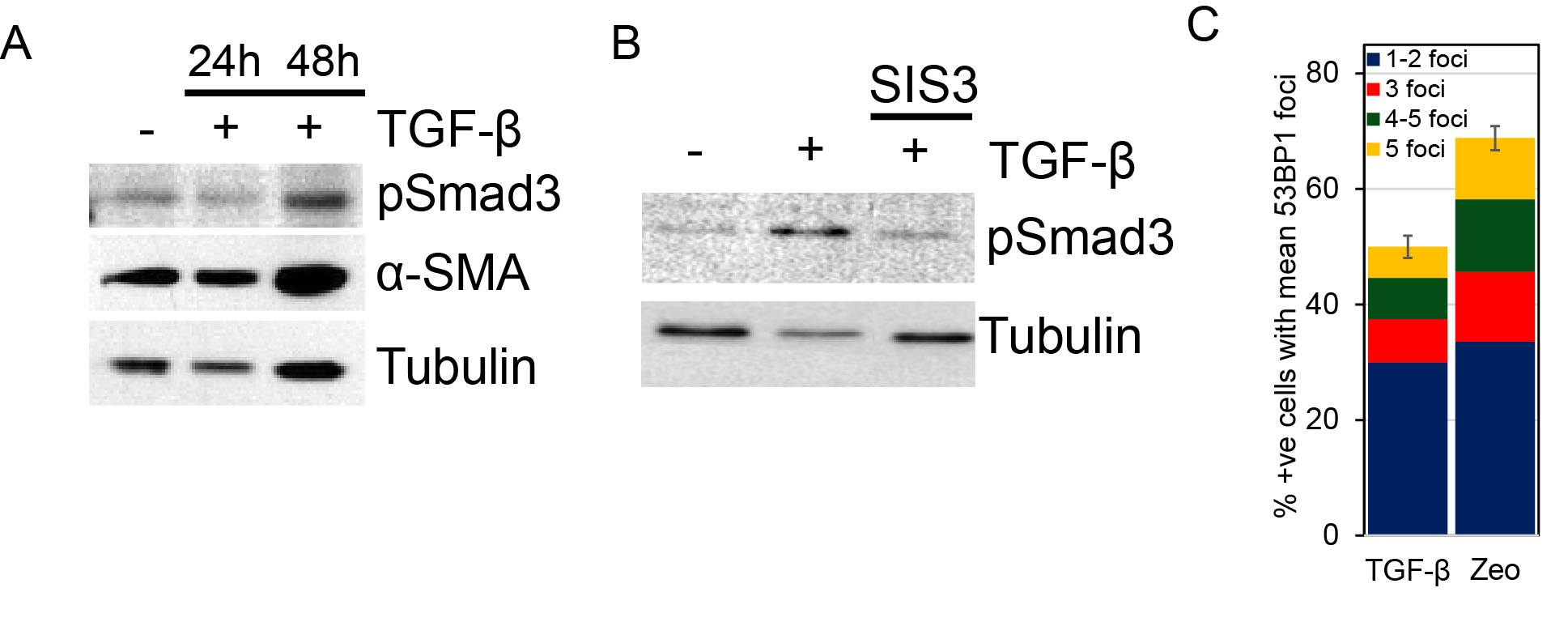


**Figure S4: Smad3 is activated in response to TGFβ1-signaling in human BJ fibroblasts**

**A** Immunoblot of cell extracts from BJ fibroblast untreated (-) or treated (+) with TGF-β1 (10 ng/ml) for a period of 24-48 hrs using antibodies against phospho-Smad3 and αSMA. γ-tubulin was used as loading control. **B** Immunoblot of cell extracts from BJ fibroblast untreated (-) or treated (+) with TGF-β1 (10 ng/ml) using antibodies against phospho-Smad3. γ-tubulin was used as loading control. SIS3: pharmacological inhibitor of Smad3 phosphorylation. **C** Percentages of 53BP1 foci positive cells in cells treated with TGF-β1 (10 ng/ml; 48h) and zeocin (4μg/ml; 4h). Frequencies of 53BP1 foci in cells is indicated by colored bars.


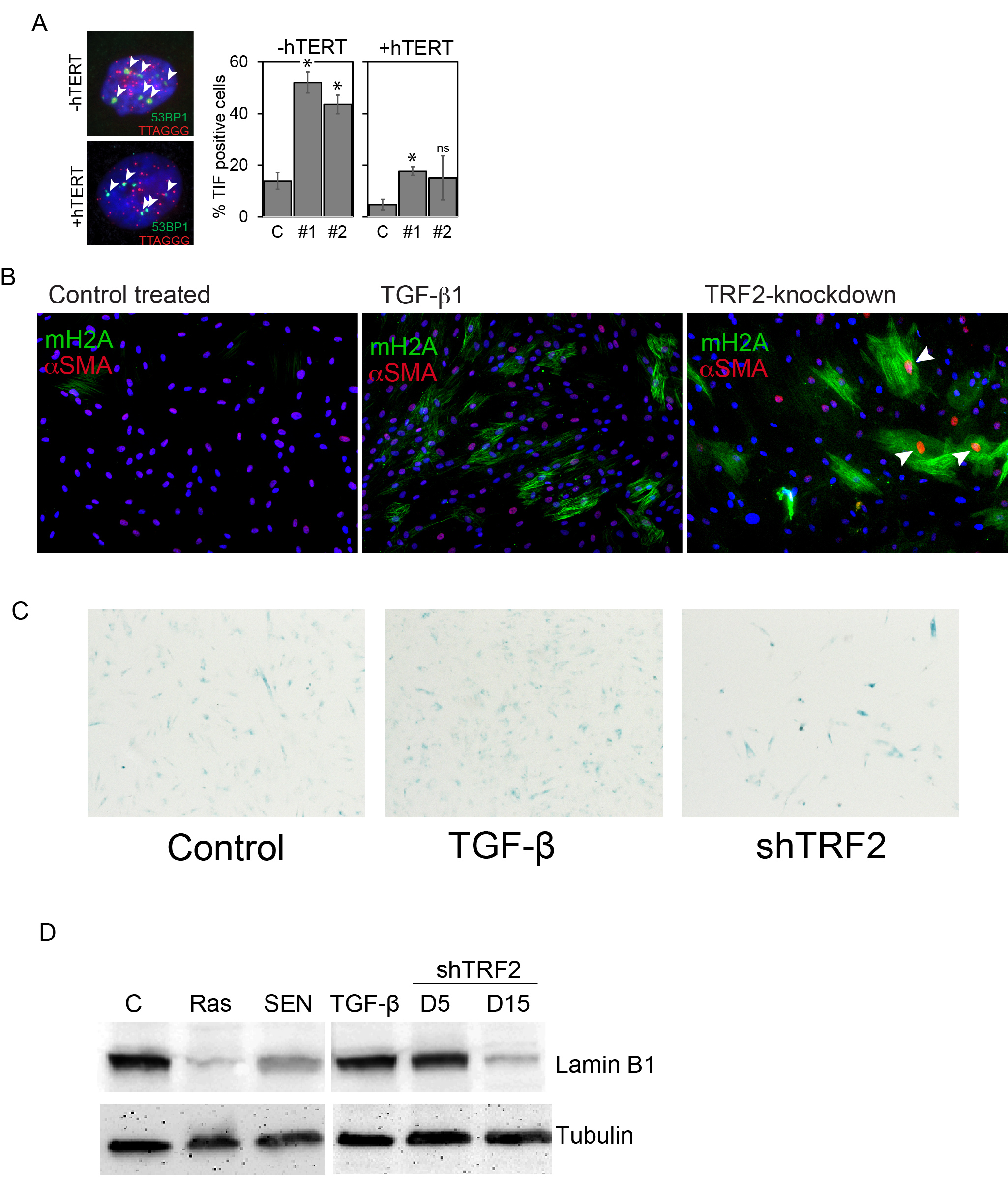


**Figure S5**. **TRF2 knockdown causes human fibroblasts to develop features of myofibroblasts and senescence. A** Normal BJ fibroblasts (-hTERT) and BJ fibroblasts overexpressing hTERT (+hTERT) in which TRF2 was knocked down using two distinct shRNA constructs (#1 and #2) were analyzed for TIF as in Figure 2C. 53BP1: green, telomere: red, DAPI: blue. Arrows indicate telomere-53BP1 colocalizations. Bar graph: quantitation of TIF positive fibroblasts as indicated. C: control knockdown vector. Error bars: +/-SD. *: p<0.05 (n=3). **B** Immunofluorescence analysis of BJ fibroblasts using antibodies against the senescence marker macroH2A (mH2A; green) and α-SMA (red) (DAPI: blue) that were either control treated (left), treated with TGF-β1 (10ng/ml) for 48h, or in which TRF2 was knocked down using shRNA (right). Scale bars: 500μm. Arrows indicate mH2A positive cells. **C** Senescence Associated-beta Galactosidase activity in fibroblasts as in B. **D** Immunoblots using antibodies against LaminB1 of early passage BJ fibroblasts (C), BJ fibroblasts treated with TGF-β1 for 48h (TGF-β), BJ fibroblasts in which TRF2 was knocked down using shRNA (shTRF2) for 5 days (D5) or 15 days (D15), H-RasG12V (Ras) senescent BJ fibroblasts, and replicatively senescent BJ fibroblasts (SEN). γ-Tubulin served as loading control.


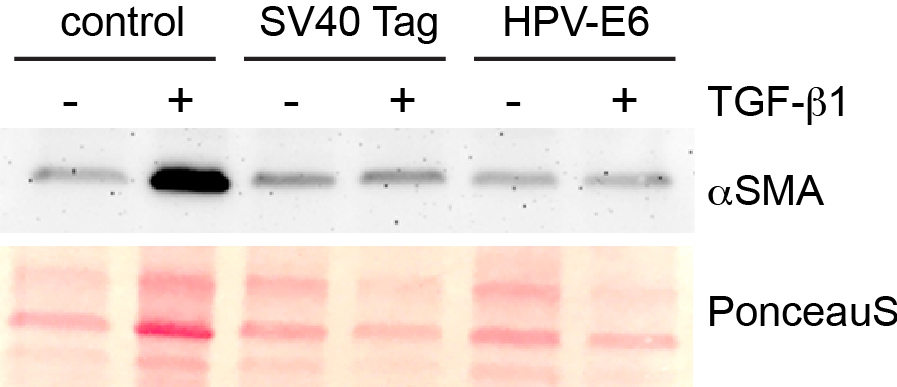


**Figure S6**. **Inactivation of p53 suppresses fibroblast to myofibroblast transdifferentiaition,** Immunoblot using antibodies against αSMA of cell extracts from BJ fibroblasts (control), BJ fibroblasts transduced with a retroviral construct expressing SV40 large T antigen (SV40 Tag), BJ fibroblasts transduced with a retroviral construct expressing HPV-E6 that were either control treated (C, -) or treated with TGFβ1 for 48h (+). PonceauS staining served as loading control.
